# 1586. Impact of Dolutegravir-based Regimen on Metabolic Syndrome and its Components in Adults with HIV in Care at Hospital Roosevelt in Guatemala City

**DOI:** 10.1093/ofid/ofad500.1421

**Published:** 2023-11-27

**Authors:** Alvaro J Mazariegos Farfán, Hugo Marroquin, Dean Ortiz, Luis Parra-Rodriguez, Andrej Spec, Johanna Meléndez, Rodolfo Pinzón, Johanna Samayoa, Carlos Mejia-Chew, Jane O’Halloran

**Affiliations:** Hospital Roosevelt, Guatemala City, Guatemala, Guatemala; Hospital Roosevelt, Guatemala City, Guatemala, Guatemala; Hospital Roosevelt, Guatemala City, Guatemala, Guatemala; Washington University School of Medicine in St. Louis, St. Louis, Missouri; Washington University in St. Louis, St. Louis, Missouri; Hospital Roosevelt, Guatemala City, Guatemala, Guatemala; Hospital Roosevelt, Guatemala City, Guatemala, Guatemala; Hospital Roosevelt, Guatemala City, Guatemala, Guatemala; Washington University in St Louis, St. Louis, Missouri; Washington University School of Medicine in St. Louis, St. Louis, Missouri

## Abstract

**Background:**

Integrase inhibitors (INSTI) are associated with weight gain, elevated glucose and lipid changes. Use of the INSTI dolutegravir (DTG) is increasing in Latin America. We assessed the impact of DTG switch on Metabolic Syndrome (MetS) and its components in people with HIV (PWH) in Guatemala.

**Methods:**

Participants with an HIV RNA < 1000 copies/ml on non-INSTI antiretroviral therapy (ART) for > 6 months who had a study visit between June 2019 and March 2020, and had complete data on MetS criteria were followed prospectively until May 2022. Those who switched to DTG-based regimen during follow up were compared to those who remained on a non-INSTI regimen. Switch participants were required to have remained on DTG for one year. We used the Latin American Diabetes Association criteria for MetS and cut-offs for waist circumference. Rates of MetS and its components were calculated at baseline and follow-up. Covariates included sex, residence, ethnicity, CD4 count, BMI, and ART. Significant covariates (p < 0.05) were included in multivariable logistic regression analyses.

**Results:**

Of 258 participants included, 82% were on efavirenz-based regimens at baseline, 52% switched to DTG (median DTG exposure of 17.5 months, interquartile range [IQR] 14.7-21.7). Overall, 91.5% remained on the same ART backbone. The MetS rate was 29.5% at baseline, and there was no between-group difference in MetS and its components. The non-switch group had a greater proportion of females, indigenous ethnicity, and a higher BMI (Table 1). MetS rate was 39.5% at follow-up (+7.4% switch group vs +13% non-switch group; p=0.39). On multivariable analysis there was no association between DTG switch and MetS (aOR 0.93; IC 0.52,1.6; p=0.78). Participants that switched to DTG were less likely to meet hypertriglyceridemia criteria (aOR 0.53; IC 0.38, 0.90; p=0.02), and more likely to meet HDL cholesterol criteria (aOR 1.93; IC 1.1,3.3; p=0.02) (Table 2).

**Figure:**
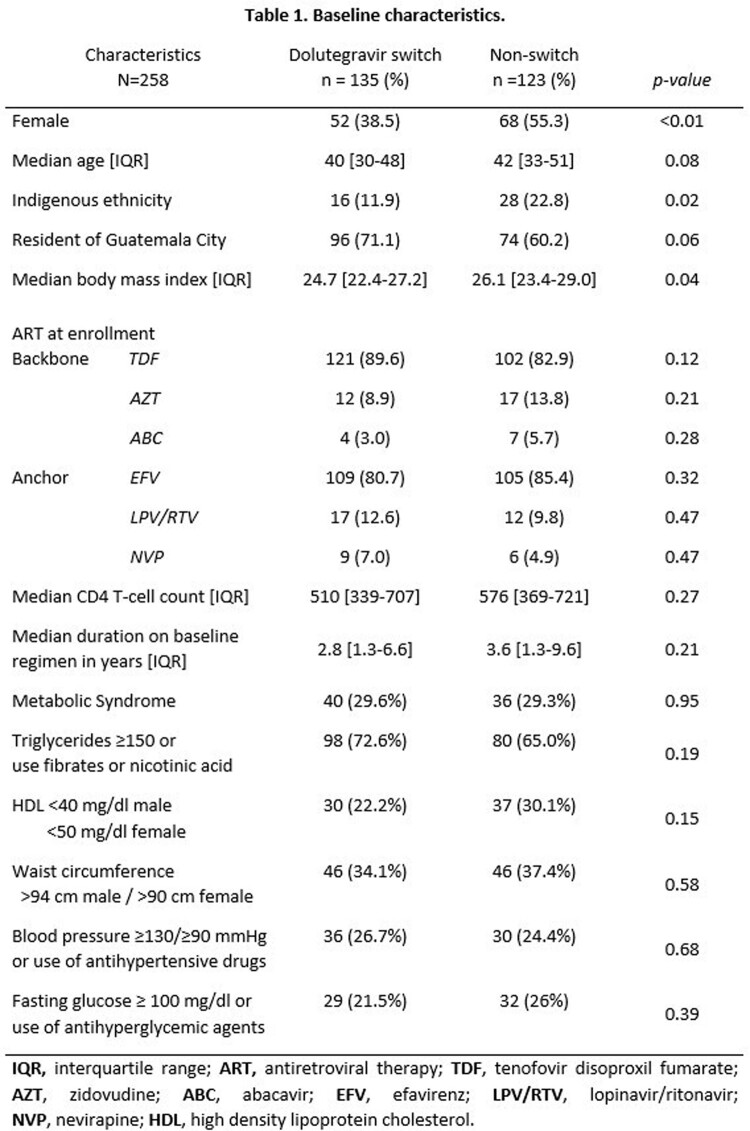

**Figure:**
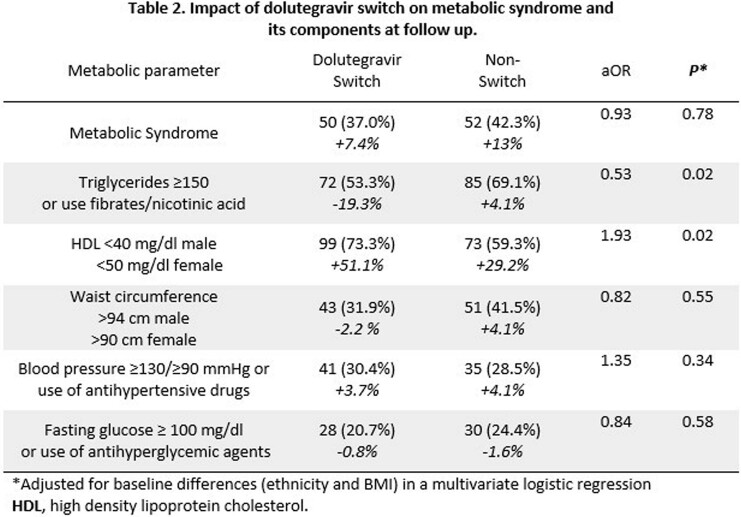

**Conclusion:**

Overall, MetS increased substantially in the follow-up period in PWH in Guatemala. We found no association between DTG switch and increased MetS. Although hypertriglyceridemia improved following DTG-switch, the potential beneficial effect might be offset by a greater reduction in HDL cholesterol, highlighting the importance of examining individual components of MetS in PWH.

**Disclosures:**

**Carlos Mejia-Chew, MD**, INSMED: Grant/Research Support|RevImmune: Grant/Research Support

